# Splitting failure in side walls of a large-scale underground cavern group: a numerical modelling and a field study

**DOI:** 10.1186/s40064-016-3214-1

**Published:** 2016-09-13

**Authors:** Zhishen Wang, Yong Li, Weishen Zhu, Yiguo Xue, Song Yu

**Affiliations:** 1 Geotechnical and Structural Engineering Research Center, Shandong University, Jinan, 250061 Shandong Province People’s Republic of China; 2 School of Civil Engineering, Shandong University, Jinan, 250061 Shandong Province People’s Republic of China

**Keywords:** Transverse isotropic constitutive model, Splitting failure, High in situ stress, Large-scale underground caverns, Numerical simulation, Field monitoring

## Abstract

Vertical splitting cracks often appear in side walls of large-scale underground caverns during excavations owing to the brittle characteristics of surrounding rock mass, especially under the conditions of high in situ stress and great overburden depth. This phenomenon greatly affects the integral safety and stability of the underground caverns. In this paper, a transverse isotropic constitutive model and a splitting failure criterion are simultaneously proposed and secondly programmed in FLAC3D to numerically simulate the integral stability of the underground caverns during excavations in Dagangshan hydropower station in Sichuan province, China. Meanwhile, an in situ monitoring study on the displacement of the key points of the underground caverns has also been carried out, and the monitoring results are compared with the numerical results. From the comparative analysis, it can be concluded that the depths of splitting relaxation area obtained by numerical simulation are almost consistent with the actual in situ monitoring values, as well as the trend of the displacement curves, which shows that the transverse isotropic constitutive model combining with the splitting failure criterion is appropriate for investigating the splitting failure in side walls of large-scale underground caverns and it will be a helpful guidance of predicting the depths of splitting relaxation area in surrounding rock mass.

## Background

In recent years, the developments of large-scale underground caverns in hydropower stations are dramatically increasing in China. A large number of underground engineering projects are being carried out, some of which are buried deep, in particular (Liu 2009; Zhu et al. 2011; Li et al. 2014). In the complex geological conditions, longitudinal splitting cracks, appearing very often in the side wall of the caverns during excavations, which can weaken the mechanical properties of the sidewall. Hibino and Motojma (1995) conducted research analyzing statistical data from a large number of monitoring supervise of 16 hydropower caverns by borehole TV and extensometer. The results showed that inside the rock wall of cavern there were opening deformation—crack propagation, joint opening, new crack generation in rock mass after excavation (Li et al. 2011). In the recent decade, the stability of some underground hydropower projects is greatly affected by this kind of splitting failures in the surrounding rock masses of underground cavern groups (Jiang 2007; Zhu et al. 2012; Xiang et al. 2011).

The splitting failure in the sidewall may destabilize the underground engineering projects (Liu 2009), as well as reducing crane beam stability (Zhang et al. 2012) and bearing capacity (Xiang et al. 2011). Moreover, other engineering hazards such as corrosion, rock bolt cables failure and cavern leakage may be induced by the splitting failure (Wei et al. 2010).

Previous studies, such as model of crack propagation or micro crack (Dias-da-Costa et al. 2011; Yerramalli and Waas 2001; Nie et al. 2015; Haeri et al. 2014) were no longer applicable when the micro-cracks in the rock masses propagated (Haeri et al. 2015) and became large parallel splitting crack groups. Therefore, it is necessary to find a more suitable constitutive model which can determine the effectiveness of the research results.

In this paper, the location, depth and the development characteristics of the splitting failures during excavations have been numerically simulated based on a transverse isotropic constitutive model combined with a splitting failure criterion at the Dagangshan hydropower station. Also an in situ monitoring study on the displacement of key points in the underground caverns was performed. The comparative analysis between numerical simulation and in situ monitoring show that the transverse isotropic constitutive model combining with the splitting failure criterion is appropriate for investigating the splitting failure in side walls of large-scale underground caverns and it will be a helpful guidance of predicting the depths of splitting relaxation area in surrounding rock mass.

## A transverse isotropic constitutive model

**Fig. 1:**
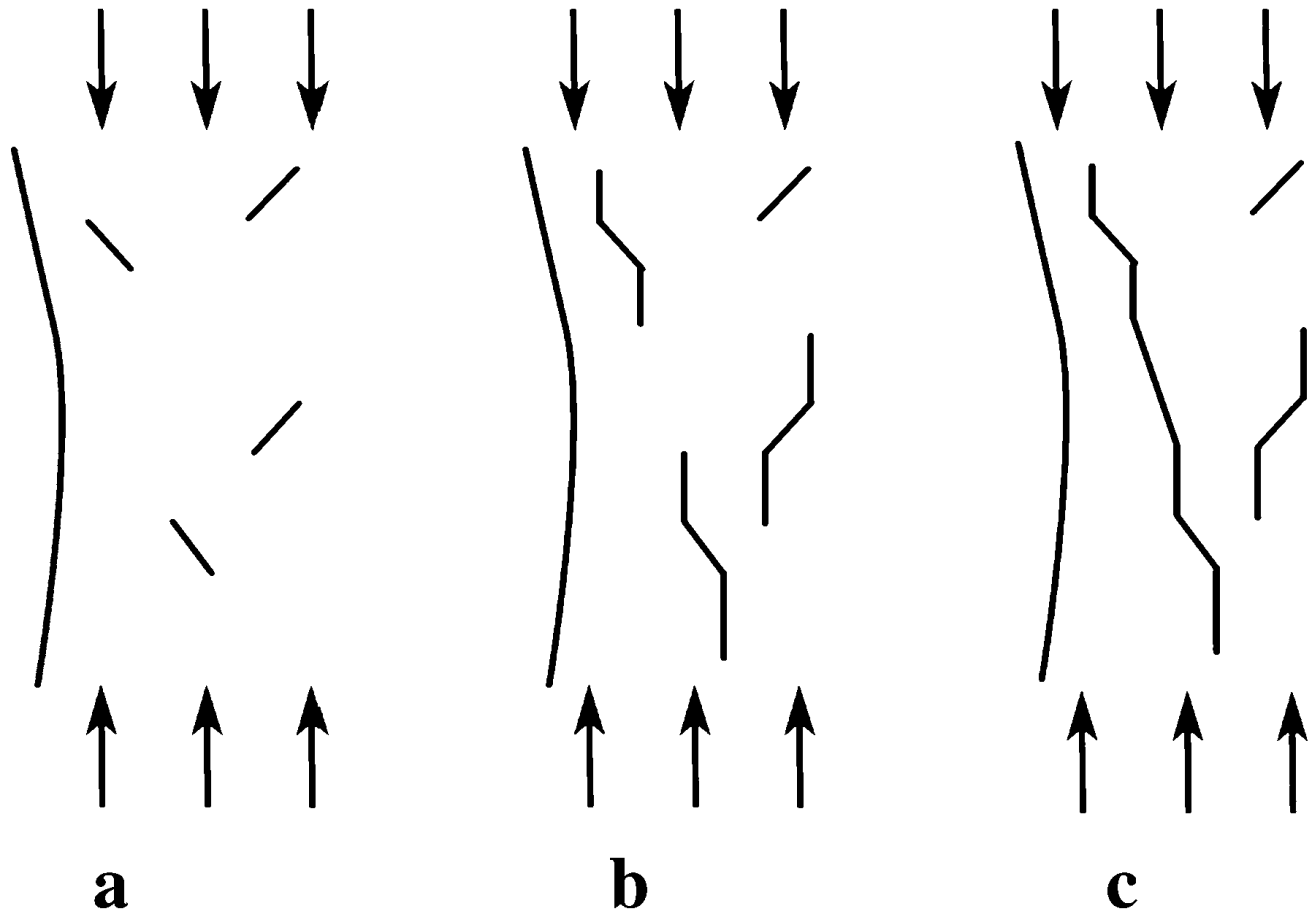
Schematic diagrams of splitting failure mode of surrounding rock: **a** The initial condition of the surrounding rock mass after the cavern excavation; **b** the extending of the crack under the continuous loading; **c** large-scale splitting failure formed

As shown in Fig. 1a, the splitting failure of underground caverns can be described as the stress of the side wall concentrated after the cavern excavations, which is caused by the similar compressed state near the excavation zone, resulting from the unloading on the other side of the cavern. Then the crack will extend with the continuous loading, depicted in Fig. 1b. When the stress state of the crack meets the cracking conditions, the crack will extend steadily along in the direction of the principal compressive stress. As the load continues to increase, with the effect of free boundary conditions and the interactions among the cracks, the crack propagation will not be stable. At this stage, the cracks growth will break out and large-scale splitting cracks will be formed as shown in the Fig. 1c.

Since the brittle characteristics of rock mass, the vertical splitting cracks of the rock masses between the caverns often appear during excavations. Therefore, the rock masses in splitting areas could be simplified to laminated model where the joint plane is perpendicular to the horizontal plane. This could be described by the transverse isotropic constitutive models (Jia et al. 2008).

**Fig. 2:**
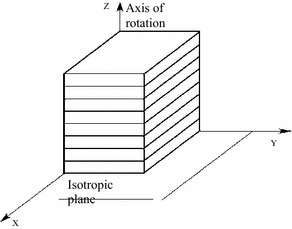
Transverse isotropic material

Transverse isotropic elastomer is composed of a series of elastic symmetry planes (Ngueyep Mambou et al. 2015; Janna et al. 2012). The physico–mechanical properties of the transverse isotropic elastomer are totally different parallel to and perpendicular to the planes of elastic symmetry. The joint plane is shown as the plane of elastic symmetry (David 2012), the material parallel to the joint plane has the same physico-mechanical properties (Aboye and Nadarajah 2012), and vertical direction has different physico-mechanical properties, as shown in Fig. 2. It is assumed that the XOY plane is an elastic plane and the elastic parameters of the transverse isotropic elastomer should meet the following conditions:

1 $$\begin{aligned} E_{x} & = E_{y} = E,\quad E_{z} = E^{{\prime }} \\ v_{xy} & = v_{yx} = v,\quad v_{zx} = v_{zy} = v^{{\prime }} \\ G_{xy} & = \frac{E}{2(1 + v)},\quad G_{yz} = G_{xz} = G^{{\prime }} \\ \end{aligned}$$

The constitutive relation of the transversely isotropic elastomer can be simplified as:

2 $$\left\{ {\begin{array}{*{20}c} {\varepsilon_{x} } \\ {\varepsilon_{y} } \\ {\varepsilon_{z} } \\ {\gamma_{xy} } \\ {\gamma_{yz} } \\ {\gamma_{xz} } \\ \end{array} } \right\} = \left[ A \right]\left\{ {\begin{array}{*{20}c} {\sigma_{x} } \\ {\sigma_{y} } \\ {\sigma_{z} } \\ {\tau_{xy} } \\ {\tau_{yz} } \\ {\tau_{xz} } \\ \end{array} } \right\} = \left\{ {\begin{array}{*{20}c} {a_{11} } & {a_{12} } & {a_{13} } & 0 & 0 & 0 \\ {} & {a_{11} } & {a_{23} } & 0 & 0 & 0 \\ {} & {} & {a_{33} } & 0 & 0 & 0 \\ {} & {} & {} & {a_{44} } & 0 & 0 \\ {} & {} & {} & {} & {a_{55} } & 0 \\ {} & {} & {} & {} & {} & {a_{55} } \\ \end{array} } \right\}\left\{ {\begin{array}{*{20}c} {\sigma_{x} } \\ {\sigma_{y} } \\ {\sigma_{z} } \\ {\tau_{xy} } \\ {\tau_{yz} } \\ {\tau_{xz} } \\ \end{array} } \right\}$$

where [*A*] is the flexibility matrix.

The parameters of flexibility matrix in Eq. (2) could be expressed as elastic constants:

3 $$\begin{aligned} a_{11} = \frac{1}{E},\quad a_{12} = - \frac{v}{E},\quad a_{13} = - \frac{{v^{{\prime }} }}{E} \hfill \\ a_{33} = \frac{1}{{E^{{\prime }} }}, \quad a_{44} = \frac{{2\left( {1 + v} \right)}}{E}, \quad a_{55} = \frac{1}{{G^{{\prime }} }} \hfill \\ \end{aligned}$$

where $E_{1} ,\mu_{1}$ are the elastic modulus and Poisson’s ratio of the transverse isotropic plane (XOY plane); $E_{2} ,\mu_{2}$ are the elastic modulus and Poisson’s ratio of the vertical transverse isotropic plane (Z axis); $G_{2}$ is the shear modulus of the vertical transverse isotropic plane (perpendicular to the XOY plane).

The various parameters in the stiffness matrix [C] can be expressed as elastic constants since the stiffness matrix and compliance matrix are mutually inverse, which is [C] = [A]^−1^.

4 $$\begin{aligned} c_{11} & = \lambda n\left( {1 - n\mu_{2}^{2} } \right),c_{12} = \lambda n\left( {\mu_{1} + n\mu_{2}^{2} } \right),c_{13} = \lambda n\mu_{2} \left( {1 + \mu_{1} } \right) \\ c_{33} & = \lambda \left( {1 - \mu_{1}^{2} } \right),c_{44} = G_{2} ,c_{66} = E/\left[ {2\left( {1 + \mu_{1} } \right)} \right] \\ \end{aligned}$$

where $$n = \frac{{E_{1} }}{{E_{2} }},\lambda = E_{2} /(1 + \mu_{1} )(1 - \mu_{1} - 2n\mu_{2}^{2} )$$

The nine independent elastic parameters have been reduced to five with the degeneration of the orthotropic to transverse isotropic elastomer. These are ${\text{E}}_{1}$, ${\text{E}}_{2}$, ${\text{G}}_{2}$, $\upmu_{1}$, $\upmu_{2}$. ${\text{G}}_{1}$ is not the independent elastic parameter, for ${\text{G}}_{1} = {\text{E}}_{1} /[2(1 +\upmu_{1} )]$. Obviously, ${\text{G}}_{1}$ is the function of ${\text{E}}_{1}$ and $\upmu_{1}$.

## A field monitoring in Dagangshan hydropower station

Dagangshan hydropower station, with a controlled area of 62, 727 km^2^, 81 % of catchment area of Dadu River, is one of the large-scale hydropower projects in the Dadu River developed recently. The location of the hydroelectric station is at the middle reach of the Dadu River in Sichuan Province and the hydropower station is approximately 40 km from the Shimian City and approximately 72 km away from Luding City.

This hydroelectric station has 4 generator units, and the capacity of each unit is 550 MW, with a total capacity of 2, 600 MW. The capacity is 114.5 billion kW h.

The water diversion and power generation system of the hydroelectric station, which includes the main power houses, transformer chamber and tailrace surge chamber, are on the left bank of the river. The three large chambers are arranged in parallel, with axis direction is NE55°, vertical buried depths are 390–520 m and horizontal buried depths are 310–530 m. The total length of the main power house is 226.58 m, the length of the crane girder is 30.80 m, and the maximum excavation height of the main engine is 73.78 m. The transformer chamber is 144 m long, 18.8 m wide and 25.6 m high. And the length, width and height of the tailrace surge chamber is 132 m, 24 m and 75.08 m, respectively.

The type of the rock surrounding the underground powerhouse is mainly fine grained biotite adamellite (γ24-1), and the surrounding rock partial interspersed with diabase. The rock masses of the plant area are massive and similar-massive structure. There are some weak structural planes such as faults f57, f58, f59, f60 and etc. through the plant caverns.

Generally, there are four groups of fracture developed in the rock masses of the plant area at the hydropower station, the length of which are 3–5 m mainly, and some are longer than 10 m individually. There are also some alterations on the surface of the fracture in groups. The steep angle fractures whose direction is nearly SN are most developed. The angle between axis of underground powerhouse, which first proposed for NE55°, and the maximum principal stress is small. According to the classification of surrounding rock, the classification of surrounding rock of main power house, transformer chamber and tailrace surge chamber is between Type II and Type III. Overall, the surrounding rocks of the plant area are stable. The results of field monitoring and the reverse calculation show that the in situ stress field in the underground powerhouse area is a superposed stress field which is comprised of tectonic stress and gravity stress. The distribution of in situ stress is affected comprehensively by tectonic stress, geological structure and topography (Zhang et al. 2011). The area can be regarded as a high in situ stress area (Zhang et al. 2009) and the maximum principal stress is up to 26.9 MPa (Li 2009).

**Fig. 3:**
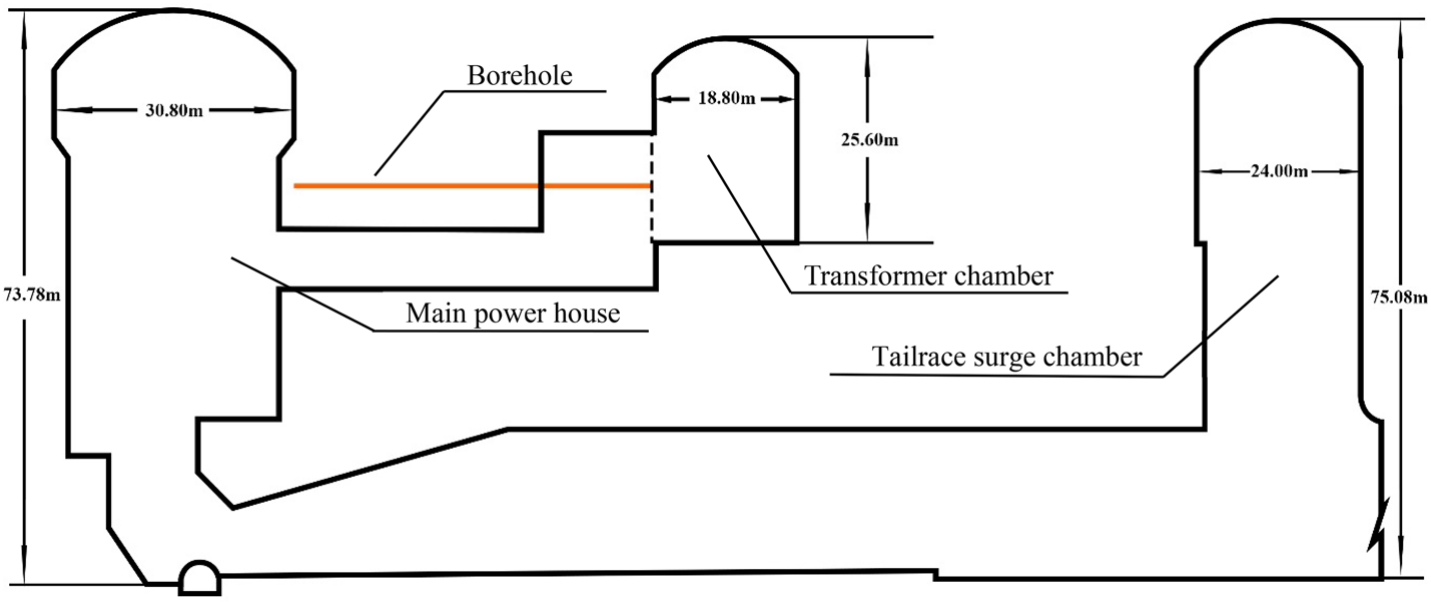
The profile of the monitoring holes

**Fig. 4:**
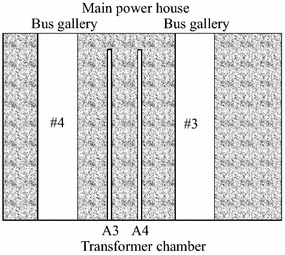
The planar graph of the monitoring hole: *A3*, *A4* are the boreholes that drilling from transformer chamber to main power house

Four pre-set horizontal monitoring holes, each to a depth of 46.5 m and diameter of 110 mm, have been constructed to obtain the complete values of the displacement and splitting zone of the downstream surrounding rock masses in the main power house during excavations. The layout of the holes is shown in Figs. 3 and 4. Among of them, the holes A3 and A4 that are 4.5 m apart and located in the rock masses between bus gallery #3 and #4 are put into a group for sliding extensometer and deformation resistivity instrument monitoring. Since the transformer chamber has been completely constructed, the drilling direction is from the upstream sidewall of transformer chamber to the main power house in order to make less interference suffered during excavations in the main power house.

Three types of monitoring methods were used in the field monitoring including the high-accuracy sliding extensometers, WDA-1 and borehole TV.

High-accuracy sliding extensometers made in Switzerland was used to measure the displacement changes of the surrounding rock mass.

WDA-1, a kind of high density resistivity meter, has been applied in the field resistivity monitoring. The length of the probe of WDA-1 is 150 cm, with two electrodes of A and M. Another pair of electrodes, N and B, are arranged at infinity. A and B generate current, while N and M monitor the generated voltage to obtain the resistivity changes of rock masses after calculation.

Using borehole TV, the camera probe is pushed into the horizontal monitoring holes to record video using spindles, by which the cracks number can be counted and recorded.

From the monitoring results of the three methods, the failure zones of the surrounding rock masses caused by excavation disturbance were determined. The depths of the splitting zones of the main power house are larger than those of the transformer chamber, since the larger size of the main power house. For the generator unit #4, the depth of the splitting failure zone of the main power house is significantly larger than that of the transformer chamber.

Due to differences in accuracy and the abilities of the anti-interference of various monitoring instruments, the results of the three kinds of monitoring methods are slightly different, but the overall trend is consistent. The results monitored by the sliding extensometers could be considered more reliable than the others for its high precision, strong anti-interference and fewer interferential factors, while the remaining two could be employed as useful supplements. According to the monitoring results and combined with the engineering experience, the depth of the splitting failure zone of the #4 generator unit is shown in Table 1.

**Table 1:** Depth of the splitting area of the generator unit #4 by field monitoring

Unit section	Generator unit #4	
Location	Downstream of the main power house	Upstream of the transformer chamber
Depth of splitting area (m)	13–15	5–6

The max displacement of the downstream sidewall of main power house is approximately 17–18 mm and that of upstream sidewall of transformer chamber is approximately 5 mm.

## Establishment of computational model combining with a splitting failure criterion

### Stress state conditions and a prediction formula of the splitting surrounding rock

Li (2007) deduced the splitting failure criterion for a fractured surrounding rock mass using a slip crack model as below:

5 $$\sigma_{1} \ge \frac{{K_{IC} \sqrt {\pi L} }}{{L(sin\theta \,cos^{2} \theta - \mu sin^{2} \theta cos\theta )}} + \sigma_{3} \frac{{\pi + (sin\theta \,cos^{2} \theta + \mu cos^{3} \theta )}}{{sin\theta \,cos^{2} \theta - \mu cos\theta \,sin^{2} \theta }}$$

where $\mu$ is the friction coefficient of initial crack, $L$ is the average length of the initial crack (m), $\theta$ is the angle between initial crack and the horizontal direction (°), $K_{IC}$ is the fracture toughness of the rock (MPa m^1/2^).

This criterion can be used to predict the depth of the splitting zone in the surrounding rock masses of underground caverns.

### Computational model and parameters

**Fig. 5:**
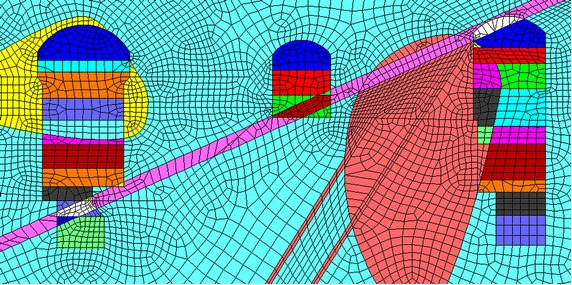
Numerical model of the generator unit #4

In this paper, a numerical model of the main power house, transformer chamber and tailrace surge chamber of generator unit #4 is established to calculate and analyze the stability of surrounding rock masses, illustrated in Fig. 5. The excavation process of the main power house is divided into eight steps from top to bottom, and the transformer chamber is divided into three steps and tailrace surge chamber is divided into seven steps.

The scope of the numerical model is as follows: upstream boundary: extending 300 m from the upstream sidewall of the main power house to upper reaches; downstream boundary: extending 300 m from the downstream sidewall of the tailrace surge chamber to lower reaches; top boundary: to ground elevation; bottom boundary: to 1120 m elevation (the elevation of the floor of the tailrace surge chamber is 920.02 m). There are a number of faults and fractured areas in the domain of the numerical model. So as to facilitate the calculation, only the faults and fracture areas which has a great influence on the three major chambers is simulated in the numerical simulation.

According to the results of field monitoring and inversion analysis, the physico-mechanical parameters of the numerical model of the generator unit #4 are shown in Table 2.

**Table 2:** Mechanical parameters of the numerical model of the generator unit #4

Rock mass classification	Bulk density (kN/m^3^)	Deformation modulus (GPa)	Friction angle (°)	Cohesion (MPa)	Poisson’s ratio	Tensile strength (MPa)
II	26.5	23.5	52.4	2.0	0.25	8.0
III	26.5	9.5	47.7	1.4	0.27	6.0
IV	26.5	2.5	38.7	0.7	0.35	4.0
II/III	26.5	16.5	50.2	1.7	0.26	7.0

### Calculation process

During numerical simulations, the first stage uses the Mohr–Coulomb model with the corresponding parameters in FLAC3D, and then proceeds to the excavation styles. After each step of excavation, the splitting fracture zone is determined by the criterion given in Eq. (5). Then, the structural model of the fracture zone is be transformed into the transverse isotropic model. The vertical direction parameters remain as the original while the horizontal ones are decreased. The main power house is divided into eight steps to be excavated. Therefore the numerical simulation was ceased after the eighth step excavation was completed.

The numerical simulation is carried out according to the following procedure:

1. The main parameters of Eq. (5) are determined according to the field rock mass condition;
2. Importing the numerical model into the FLAC3D;
3. Writing the Eq. (5) with the parameters into FLAC3D to gauge the stress field of the numerical model, and the parts that meet the criterion are considered that the rock mass in these positions has a splitting failure. These sections of the constitutive model are changed into transverse isotropic constitutive model.
4. Calculating the stability of the caverns during excavations.

**Fig. 6:**
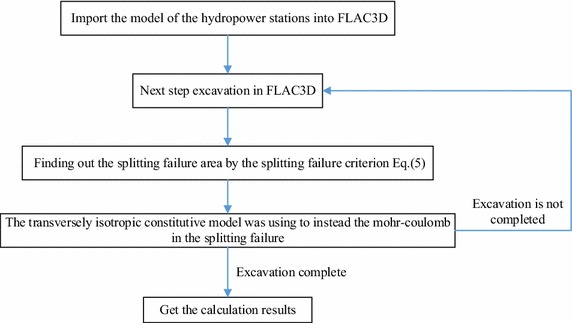
A calculation flow chart

The flow chart is shown in Fig. 6.

## Analysis of calculation results

### General analysis of the splitting failure of surrounding rock mass

According to the exception in field and some experiences as well as inversion analysis, the main parameters of the Eq. (5) are adopted as follows:

$$\upmu = 0.2,\quad\uptheta = 40^{^\circ } ,\quad {\text{K}}_{\text{IC}} = 0.84\,{\text{MPa}}\,{\text{m}}^{1/2} ,\quad {\text{L}} = 5\,{\text{m}}$$

**Fig. 7:**
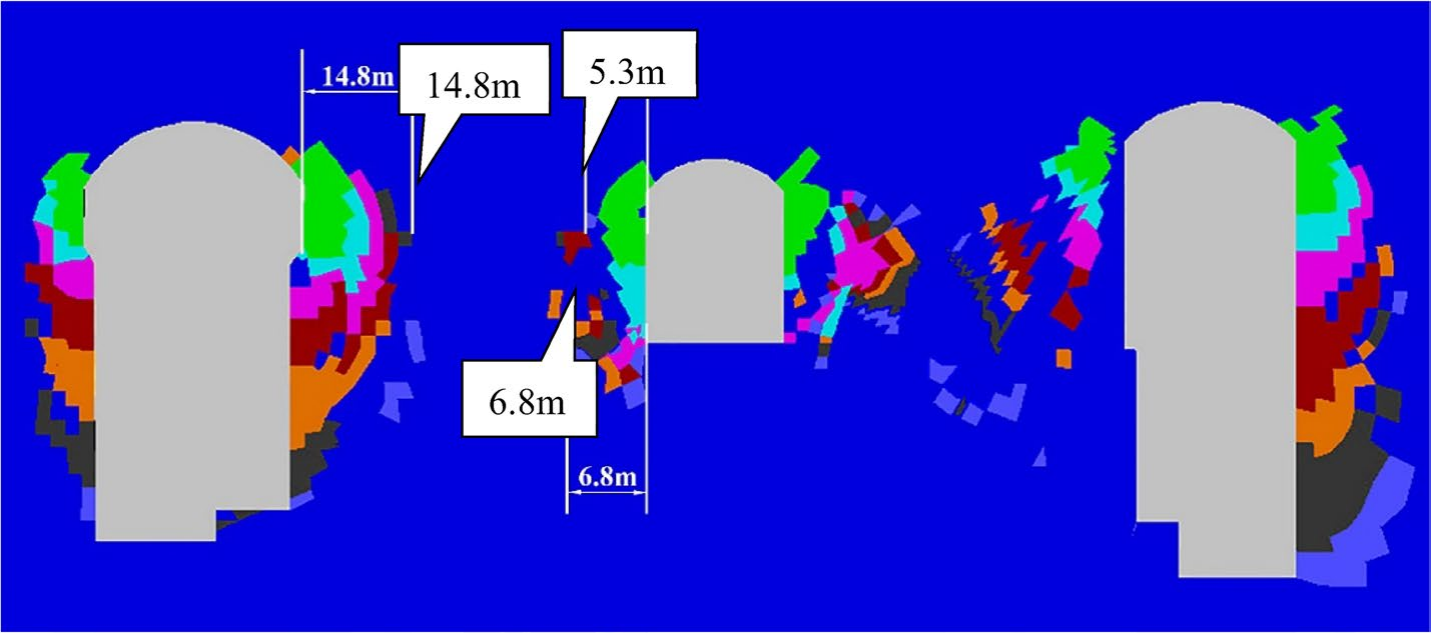
The splitting area of the generator unit #4 after eight-step excavation

Firstly, taking the parameters into the Eq. (5) and principal stress of the surrounding rock masses has been achieved, then calculating by FLAC3D. After that, the splitting area of the generator unit #4 after eight-step excavation could be gotten and shown in Fig. 7.

According to Fig. 7, the depth of the splitting zone of the underground caverns has been gradually increased with the excavation steps. The depth of splitting zone of the downstream sidewall of the main power house is slightly larger than that of the upstream sidewall of the main power house and that of the upstream sidewall of the transformer chamber. The depth of splitting failure zone between the transformer chamber and tailrace surge chamber is relatively large due to the influence of the fault. The maximum depth of the splitting zone on the downstream sidewall of the main power house is approximately 14.8 m, while the average of that is 6.8 m after the eighth excavation is completed. The calculated results are almost consistent with the monitoring results shown in Table 1.

### Deformation analysis of surrounding rock mass

**Fig. 8:**
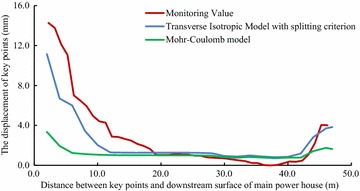
The displacement curves of the key points of main power house after six-step excavation

**Fig. 9:**
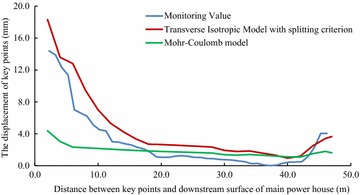
The displacement curves of the key points of main power house after seven-step excavation

**Fig. 10:**
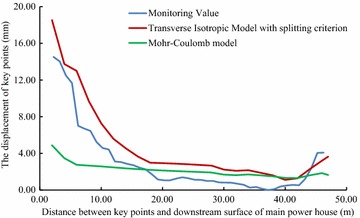
The displacement of the key points of main power house after eight step excavation

For validating the calculation results by FLAC3D, the displacements of some key points in the model, shown in Figs. 3 and 4, have been compared with the displacements obtained by field monitoring at the same positions as in the model, shown in Figs. 8, 9 and 10.

Since the large size of the main power house, significant disturbing effect on the surrounding rock is caused by excavations of the main power house. Therefore, the displacement of the key points near the downstream surface of the main power house is much larger than that near the upstream surface of the transformer chamber. According to the field monitoring, the maximum displacement of surrounding rock, 18.53 mm, as measured near the surface of the downstream sidewall of the main power house. The displacement of surrounding rock masses decreases with the distance from the surface of the downstream sidewall of the main power house to the key points. The displacement curve has a turning point when the distance between the monitoring points and the main workshop is approximately 15 m. After that, the descending of displacement is becoming insignificant, whereas the displacement is approaching a constant. The displacement of surrounding rock masses begins to be gradually increased when the monitoring points from the surface of the downstream sidewall of the main power house is approximately 41 m that is 5 m from the surface of the upstream sidewall of the transformer chamber. The displacement curve reaches a peak when the monitoring point from the surface of upstream sidewall of the transformer chamber is approximately 2 m away. After the completion of excavation for the eighth step of main power house, the maximum displacement measured at this position is 4.08 mm.

According the comparison of the numerical results, the displacement curves of the key points are relatively flat when only the Mohr–Coulomb model is employed in numerical simulation, and the calculated results of the key points that near the downstream sidewall of the main power house is far less than the monitoring values, approximately 30 % of the later ones, indicating that cavern deformation caused by the splitting failure exists in the rock masses of the downstream sidewall of the main power house, which can be concluded the isotopic Mohr–Coulomb model is inappropriate for numerical modelling in splitting failures of rock mass. On the other hand, when the model of the splitting failure area adopts the transverse isotropic constitutive model, the obtained numerical results are close to the monitoring ones, and the actual displacement can be reflected.

Similar with the monitoring result, it can be seen from the displacement curve, calculated using the transverse isotropic constitutive model, that there are two turnings points in this curve, which appear at the points, 15 and 41 m from the surface of downstream sidewall of the main power house, respectively. The depth of the splitting zone in the rock mass on upstream side wall of the transformer chamber is approximately 5 m. By comparison the displacement curve that is obtained using transverse isotropic constitutive model is very similar to the monitoring curve.

### Analysis of model calculation

**Fig. 11:**
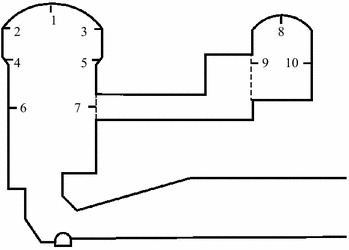
Distribution of key points of the calculating model in the numerical simulation

**Fig. 12:**
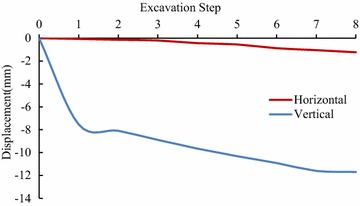
The displacement curves of the key points #1 of the main power house

**Fig. 13:**
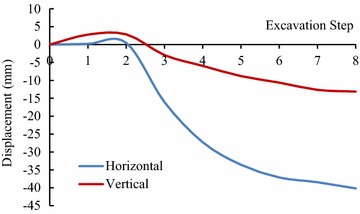
The displacement curves of the key points #5 of the main power house

**Fig. 14:**
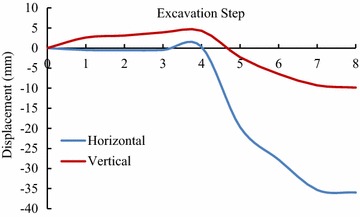
The displacement curves of the key points #7 of the main power house

**Fig. 15:**
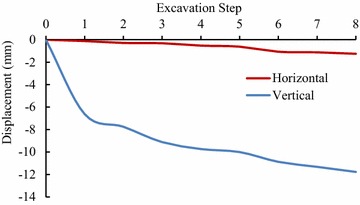
The displacement curves of the key points #8 of the transformer chamber

**Fig. 16:**
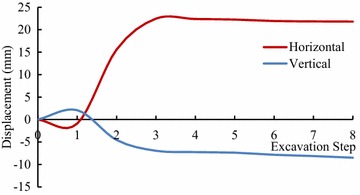
The displacement curves of the key points #9 of the transformer chamber

In order to make better understandings of the deformation and stress characteristics of each of the units, the representative key points which are on the vault, rock-bolt crane girder and the surface on the upstream sidewall of main power house and transformer chamber were selected, as well as on the downstream sidewall, as shown in Fig. 11. The extracted displacement data of each key point after calculation in FLAC3D, is shown in Figs. 12, 13, 14, 15 and 16.

After the excavations, the direction of the main deformation of the key points is inside the surrounding rock to the excavating surface, and the deformation of the key point on the vault is mainly vertical, and is horizontal when the key point was on the sidewall. The displacement of the key point of the downstream side wall of the main powerhouse is larger than that of the upstream side wall at the same altitude in unit #4. The maximum displacement of the key points of the downstream side wall of the main plant of the unit #4 is the key point #5 whose horizontal displacement is 35.97 mm after eight-step excavation. The vertical displacement of the vault of the main power house after one-step excavation is 7.54 mm, and that reaches 11.70 mm after eight-step excavation. The variation of displacement of the key points of transformer chamber is similar to the main power house. The vertical displacement of the vault of the main power house is 6.62 mm after the one-step of excavation and increases to 11.77 mm. The horizontal displacement of the upstream sidewall of transformer chamber is 21.80 mm after the eight-step excavation, which is larger than that of the downstream sidewall.

## Summary and conclusion

1. Field monitoring has been taken in generator unit #4 of Dagangshan Hydropower Station. Two monitoring holes has been constructed, and the splitting phenomenon of the rock masses between the main power house and transformer chamber has been monitored through the borehole TV, sliding extensometer and WDA-1 deformation resistivity instrument. According to the displacement monitoring results, the displacement of the rock masses decreases with the distance between the main power house and transformer chamber increasing at the beginning, and when the distance is over 41 m, the displacement is increasing again. The maximum displacement near the main power house is 18.53 mm (2 m away from the surface of the downstream sidewall of the main power house), and that near the transformer chamber is 4.08 mm (5 m away from the surface of the upstream sidewall of the transformer chamber). The monitoring result also shows that the depth of the splitting zone of downstream sidewall of main power house is larger than that of upstream sidewall. And the depth of the downstream sidewall of the main power house is approximately 13–15 m, and that of the upstream sidewall of the transformer chamber is approximately 5 m.
2. A numerical model of generator unit #4 with main power house, transformer chamber and tailrace surge chamber has been established and a transverse isotropic constitutive model and a splitting failure criterion are simultaneously employed to calculate the splitting zone of the surrounding rock masses. The maximum depth of the splitting area of the downstream sidewall of the main power house by calculation is approximately 14.8 m and the average of that of the upstream sidewall of the transformer chamber is approximately 6.8 m, which are similar with the monitoring values. By comparing the calculated and actually monitored displacement curve, it can be concluded that the displacement curve calculated with transverse isotropic constitutive model is more consistent with the actual field monitoring curve. In this way, the employment of the transverse isotropic constitutive model could numerically simulate splitting zones in brittle surrounding rock mass under high in situ stress.
3. The calculated displacement by the new model which considering the opening deformation is three times larger than that obtained by traditional elasto-plastic constitutive model. The results calculated by the new model with the transverse isotropic model are more closed to the monitoring values.
4. By analyzing the displacement curve, it can be seen that the curve has two inflections when the distances between the key points and the surface of the downstream sidewall of the main power house are 15 and 41 m, respectively, which means the depth of the splitting area of the downstream sidewall of the main power house is approximately 15 m and that of the upstream sidewall of the transformer chamber is approximately 5 m. The results show that the calculated curve is consistent with the field monitoring result.
